# Prospective observational study and mechanistic evidence showing lipolysis of circulating triglycerides worsens hypertriglyceridemic acute pancreatitis

**DOI:** 10.1172/JCI184785

**Published:** 2024-11-07

**Authors:** Prasad Rajalingamgari, Biswajit Khatua, Megan J. Summers, Sergiy Kostenko, Yu-Hui H. Chang, Mohamed Elmallahy, Arti Anand, Anoop Narayana Pillai, Mahmoud Morsy, Shubham Trivedi, Bryce McFayden, Sarah Jahangir, Christine L.H. Snozek, Vijay P. Singh

**Affiliations:** 1 Department of Medicine,; 2 Department of Clinical Trials and Biostatistics,; 3 Department of Laboratory Medicine and Pathology, and; 4 Department of Biochemistry and Molecular Biology, Mayo Clinic, Arizona, USA.

**Keywords:** Gastroenterology, Lipoproteins

## Abstract

**BACKGROUND:**

While most hypertriglyceridemia is asymptomatic, hypertriglyceridemia-associated acute pancreatitis (HTG-AP) can be more severe than AP of other etiologies. The reasons underlying this are unclear. We thus examined whether lipolytic generation of nonesterified fatty acids (NEFAs) from circulating triglycerides (TGs) could worsen clinical outcomes.

**METHODS:**

Admission serum TGs, NEFA composition, and concentrations were analyzed prospectively for 269 patients with AP. These parameters, demographics, and clinical outcomes were compared between HTG-AP (TGs >500 mg/dL; American Heart Association [AHA] 2018 guidelines) and AP of other etiologies. Serum NEFAs were correlated with serum TG fatty acids (TGFAs) alone and with the product of TGFA serum lipase (NEFAs – TGFAs × lipase). Studies in mice and rats were conducted to understand the role of HTG lipolysis in organ failure and to interpret the NEFA-TGFA correlations.

**RESULTS:**

Patients with HTG-AP had higher serum NEFA and TG levels and more severe AP (19% vs. 7%; *P* < 0.03) than did individuals with AP of other etiologies. Correlations of long-chain unsaturated NEFAs with corresponding TGFAs increased with TG concentrations up to 500 mg/dL and declined thereafter. However, NEFA – TGFA × lipase correlations became stronger with TGs above 500 mg/dL. AP and intravenous lipase infusion in rodents caused lipolysis of circulating TGs to NEFAs. This led to multisystem organ failure, which was prevented by pancreatic TG lipase deletion or lipase inhibition.

**CONCLUSIONS:**

HTG-AP is made severe by the NEFAs generated from intravascular lipolysis of circulating TGs. Strategies that prevent TG lipolysis may be effective in improving clinical outcomes for patients with HTG-AP.

**FUNDING:**

National Institute of Diabetes and Digestive and Kidney Diseases (NIDDK, NIH) (RO1DK092460 and R01DK119646); Department of Defense (PR191945 under W81XWH-20-1-0400); National Institute on Alcohol Abuse and Alcoholism (NIAAA), NIH (R01AA031257).

## Abstract 2

Admission sera of 269 AP patients showed circulating triglycerides are hydrolyzed to injurious non-esterified fatty acids (NEFA) that cause severe pancreatitis. This was verified experimentally.

## Graphical abstract

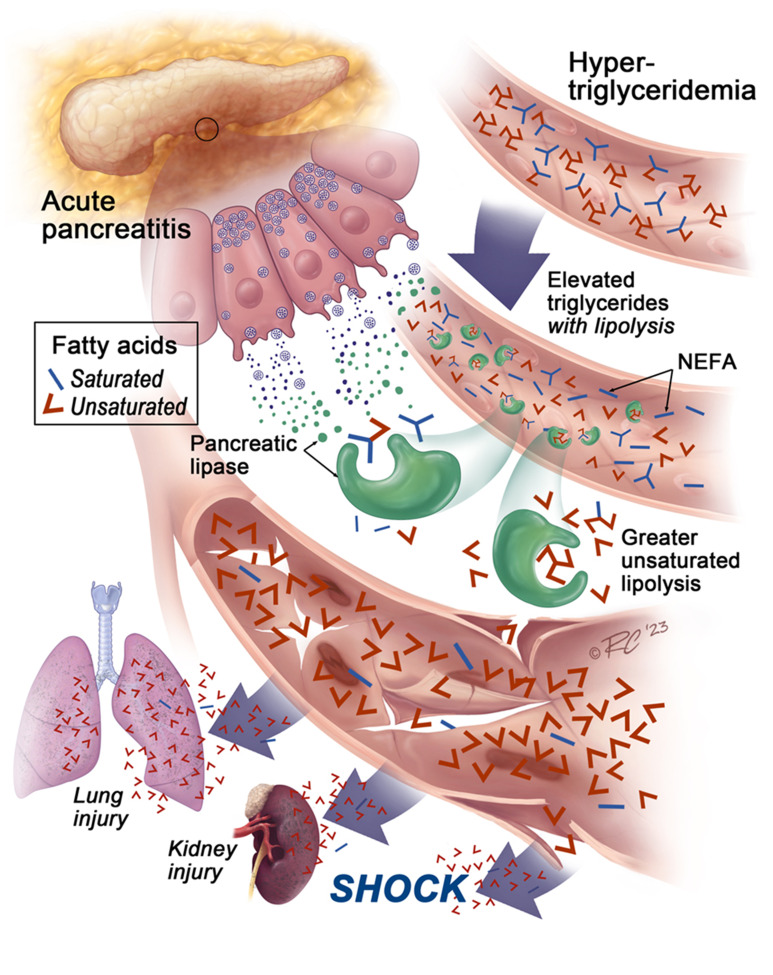

## Introduction

Acute pancreatitis (AP) is a common gastrointestinal disorder. The incidence of hypertriglyceridemia-associated AP (HTG-AP) has been increasing, with recent reports showing it to comprise up to 30% of AP cases (1, 2). Several meta-analysis show that HTG-AP increases the risk of persistent organ failure and severe AP (3–6). Even modest increases in triglycerides (TGs) above 100–200 mg/dL (2) increase the risk (2, 7) of severe AP. Several studies, including those by Nawaz et al. (8), Pascula et al. (2), and Wan et al. (7), show that severe HTG-AP may occur even when the hypertriglyceridemia is associated with a biliary or other AP etiology. Severe AP requires life support (9), increases the length of hospitalization (9), the burden on health care (9), and mortality (10, 11). However, the mechanisms behind these observations are unclear.

**Figure 1:**
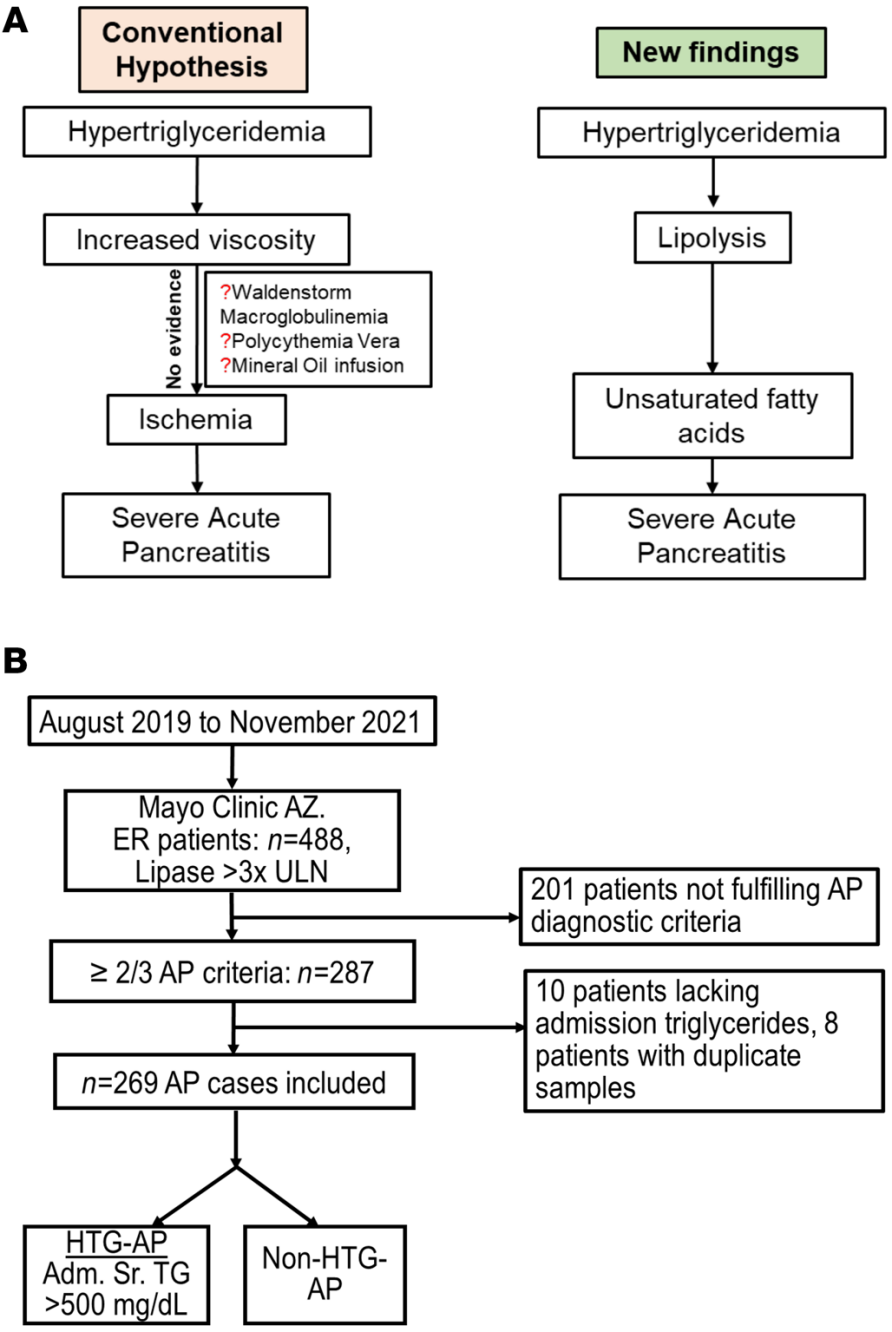
Flowcharts comparing the conventional hypothesis to the new findings and showing the design for patient selection in the current study. (**A**) Schematic of conventional hypothesis of severity of HTG pancreatitis compared with new findings. (**B**) Flowchart of patient selection for the study population of patients with AP and classification as HTG-AP versus non–HTG AP.

Serum TGs of 100–200 mM have been noted in human HTG (12, 13), and an increase in viscosity is assumed to cause AP in HTG. However, hyperviscosity-induced pancreatic ischemia seems an unlikely mechanism for AP severity (Figure 1A), since there is no evidence that hyperviscosity syndromes like Waldenstrom macroglobulinemia and polycythemia vera cause AP (14) or worsen its severity. Additionally, while perfusion of an ex vivo–isolated pancreas with TGs caused pancreatic necrosis, mineral oil infusion did not, despite its higher viscosity (15).

There is strong evidence to support the theory that unsaturated fatty acids (UFAs) cause severe AP, e.g., Sztefko et al. showed that patients developing necrotizing AP had significantly higher serum unsaturated nonesterified fatty acids (NEFAs) on admission compared with edematous AP (16). Similarly, Domschke et al. found that necrotizing AP with complications had higher serum NEFA levels on admission versus those without complications (17), and Phillips et al. showed that patients with severe AP had elevated serum unsaturated NEFAs (18). The unsaturated NEFAs linoleic acid (C18:2) and oleic acid (C18:1) cause lung injury, shock, renal failure (19, 20), and infections (21) during AP. Such organ failure may result from UFAs being released from unsaturated TG (22) breakdown in visceral fat during AP (23). Saturation interferes with the hydrolysis of unsaturated TGs (23). This is due to unsaturated TG fatty acids (TGFAs) improving the “fit” of TGs into the catalytic pocket of pancreatic lipases that hydrolyze them, as detailed elsewhere (23, 24). However, whether unsaturated TGFAs in the circulation are more likely to be hydrolyzed and released as NEFAs during HTG-AP is unknown.

TGs have 3 long-chain (≥16 carbon length) FAs (i.e., TGFAs) covalently esterified to a glycerol backbone (23). The principal long-chain NEFAs comprising the serum NEFA pool (25) are stearic acid (S) (C18:0); palmitic acid (P) (C16:0); palmitoleic acid (PO) (C16:1); oleic acid (O) (C18:1); and linoleic acid (L) (C18:2). Cumulatively, these 5 NEFAs comprise 90%–95% of the circulating NEFA pool, of which unsaturated ones comprise 55%–75% (25, 26). Serum TGs are normally in the below 2 mM range (<150 mg/dL), and serum NEFAs are normally 0.1–0.5 mM (27), increasing to the 0.6–2.0 mM range in AP (17, 21).

While previous studies have associated severe AP with unsaturated NEFAs (16, 18) and higher total serum TGs, there are scarce data comparing the composition of circulating TGs with NEFAs during AP. Under normal states, there is a weak-to-moderate strength relationship between the composition of NEFAs and TGs based on TGs being synthesized from NEFAs, referred to as the Kennedy pathway (28). It is, however, unclear whether breakdown of TGs to NEFAs can occur in the circulation as a result of AP. If so, this can explain the increased severity during HTG-AP, especially if the TGs and NEFA are unsaturated.

On the basis of the above findings and previous studies showing an association between HTG-AP or elevated unsaturated NEFAs and severe AP (2, 7, 8, 10, 11), we aimed to study whether HTG-AP had increased intravascular lipolysis of TGFAs to NEFAs, whether this was associated with severe AP (19, 21) and the relationship of this to FA unsaturation. Hence, we compared the association between serum NEFAs and serum TGFAs and studied if this was strengthened by the serum lipase increase in HTG-AP. We chose serum TG levels above 500 mg/dL to define HTG-AP based on the definition of severe hypertriglyceridemia (29) in AHA 2018 guidelines.

## Results

### Patients with HTG-AP have high serum TGs, serum NEFAs, and worse clinical outcomes.

Between August 2019 and November 2021, 488 patients presented to the emergency room (ER) of Mayo Clinic Arizona with lipase levels of more than 3 times the upper limit of normal (ULN). Of these, 201 patients were excluded, as they did not fulfill the diagnostic criteria of AP. A flowchart showing the selection of the study population is shown in Figure 1B. Of the 287 patients with AP, 18 individuals were excluded because there were no admission TG measurements or their serum samples were duplicates. Based on chart review, 27 patients had HTG-AP and 242 patients had non–HTG-AP. As shown in Table 1, age, sex distribution, BMI, diabetes status (data not shown), race, median of abdominal pain duration, and time from sample collection to processing were similar between the patients with HTG and the non–HTG-AP patients. The HTG-AP group had fewer individuals with alcoholic AP (3) and biliary AP (1) than did the non–HTG-AP group (54 and 48, respectively; *P* < 0.001). Eight patients with HTG-AP had documented hyperlipidemia before admission for AP. Six of these were on a statin, among whom 4 were also on fenofibrate, and 1 was on gemfibrozil. Only 1 patient with HTG-AP was suspected to have a genetic cause, but this was not confirmed.

We first compared serum TGs and NEFAs among the patients. As shown in Table 1, patients with HTG-AP had significantly elevated median serum TG levels [730 mg/dL (IQR: 561–1,064) or 8.5 mM (6.6–12.5) versus 161 mg/dL (IQR: 111–238) or 1.9 mM (1.3–2.8) in non–HTG-AP patients; *P* < 0.0001]. Patients with HTG-AP had significantly higher admission serum NEFAs of 0.95 mM (0.6–1.5 mM) compared with non–HTG-AP patients, who had median NEFAs of 0.61 mM (0.37–0.87 mM; *P* < 0.0001). All the principal long-chain NEFAs were increased in the patients with HTG-AP. These included palmitic acid, its unsaturated product palmitoleic acid, and also stearic acid, oleic acid, and linoelic acid.

On comparing clinical outcomes, patients with HTG-AP had significantly higher organ failure rates (30% vs. 10%; *P*:0.02) and of severe AP (19% vs. 7%; *P*:0.03) compared with non–HTG-AP patients. We observed no difference in early severity (i.e., within the first week) (30) between the 2 groups (3 of 5 in HTG-AP vs. 8 of 16 in the non–HTG-AP group; *P* = 1.0). Fifteen of 153 (9.8%) males, and 6 of 116 females (5.1%) developed severe AP (*P* = 0.18). Patients with HTG-AP also had higher intensive care unit (ICU) admission rates (26% vs. 8%; *P*:0.003) and median length of stay (5 vs. 3 days; *P*:0.0002) compared with non–HTG-AP patients. Two of the 27 patients with HTG-AP had pancreatic necrosis compared with 13 of 242 non–HTG AP patients (*P* = 0.63).

We thus went on to look for evidence supporting lipolytic generation of NEFAs during HTG-AP.

### Lipolysis of circulating TGs by pancreatic lipase generates injurious unsaturated NEFAs.

**Figure 2:**
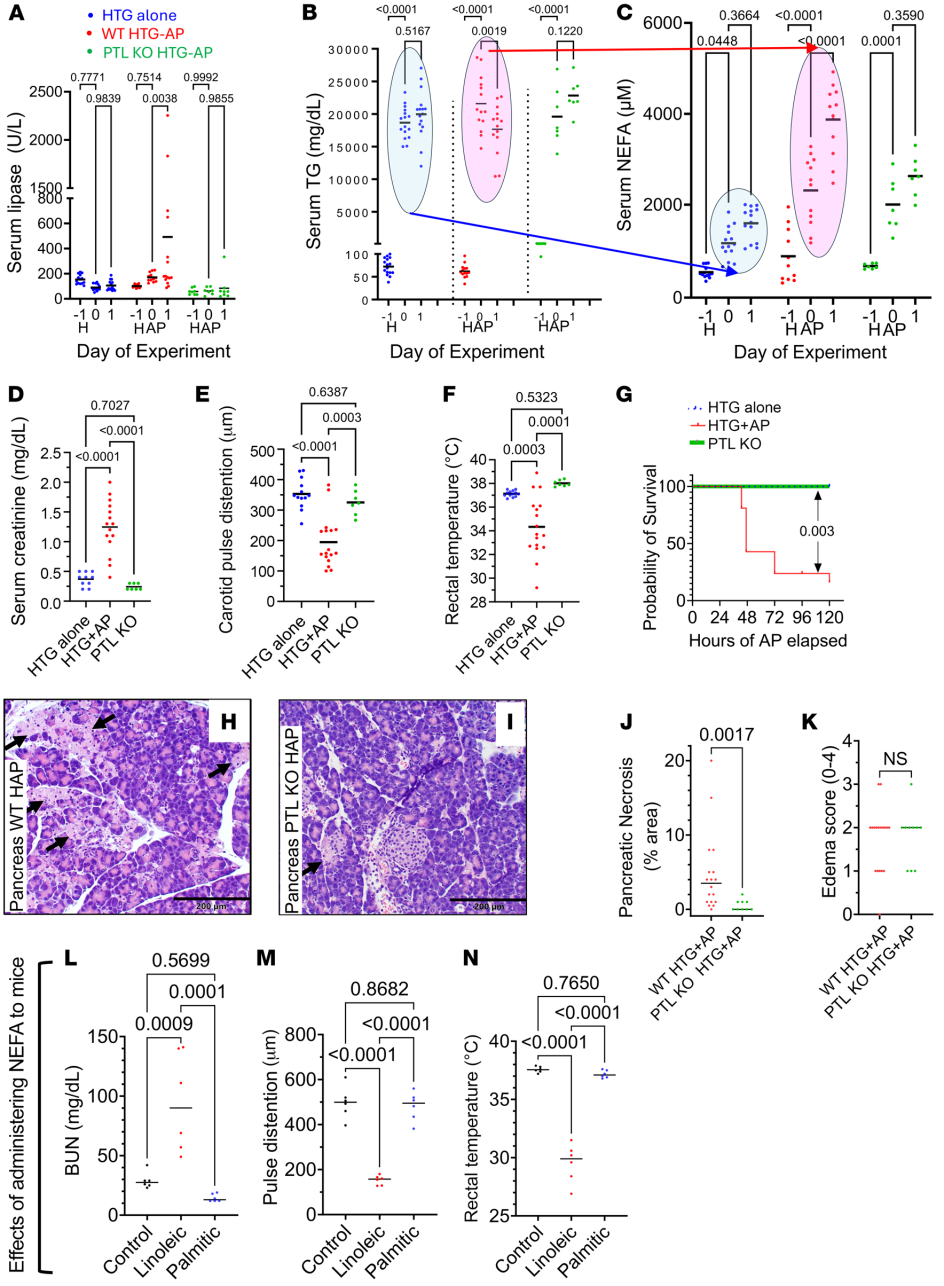
Parameters of mice with HTG alone or HTG-AP, those given NEFAs, and in vitro studies using pancreatic acini. (**A**–**H**) Mice were given poloxamer-407 on day –1 to induce HTG (H) alone (blue). HTG-AP (HAP) was induced on day 0 (i.e., 1 day after poloxamer-407) in C57bl/6 WT mice (red) or pancreatic TG lipase–KO mice (PTL KO) (green dots). Dot plots with means are shown for serum lipase (**A**), serum TGs (**B**), and serum NEFAs (**C**) for the days indicated on the *x* axis. Serum creatinine at necropsy (**D**), along with carotid pulse distention (**E**) and rectal temperature (**F**) recorded before euthanasia are shown. (**G**) Comparison of the survival curves for the 2 groups with *P* values based on the log-rank (Mantel-Cox) test. (**H** and **I**) Images of H&E-stained pancreatic sections from WT mice (**H**) and PTL-KO mice (**I**) with HAP. Scale bars: 200 μm. (**J** and **K**) Dot plots showing pancreatic necrosis (**J**) and pancreatic edema (**K**) in WT and PTL-KO mice with HAP. (**L**–**N**) Dot plots with means comparing the effects of administering linoleic acid (red dots) or palmitic acid (blue dots) on serum BUN levels at necropsy (**L**), along with carotid pulse distention (**M**) and rectal temperature (Temp) (**N**) recorded before euthanasia. P values were determined by 2-way ANOVA was done (**A**–**F**), log-rank (Mantel-Cox) test (**G**), Mann-Whitney *U* test (**J**), 2-tailed Student’s *t* test was done (**K**), and ordinary 1-way ANOVA (**L**–**N**).

**Figure 3:**
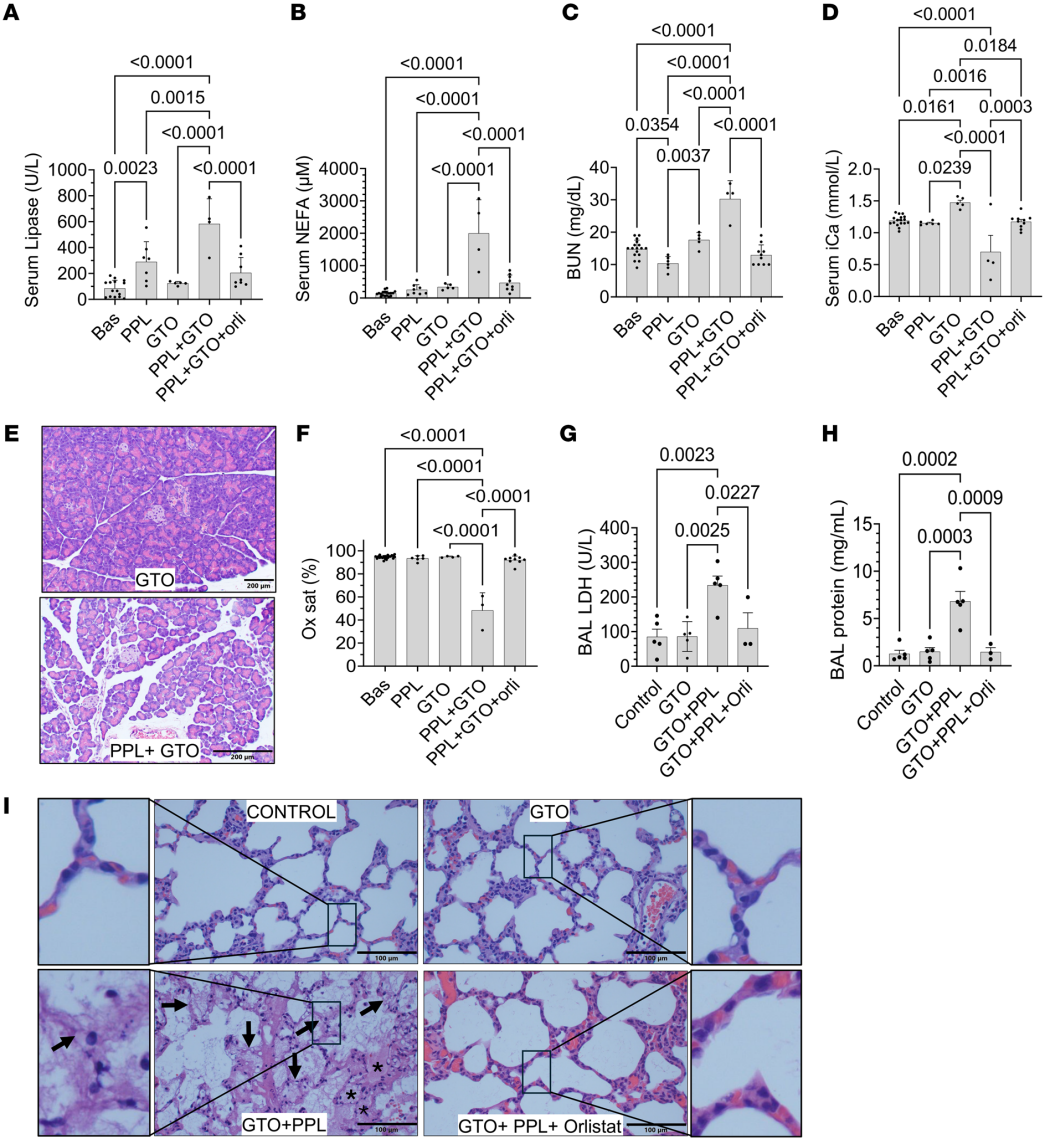
Effect on rats of intravenous infusion of TGs versus infusion of TGs with PPL, with or without the lipase inhibitor orlistat. Bar graphs (with SD and individual values) comparing the effects of infusion of PPL; TG (GTO); PPL plus TG; and PPL plus TG, plus the lipase inhibitor orlistat at baseline (Bas) and after infusion. Parameters are serum lipase (**A**), NEFAs (**B**), BUN (**C**), and ionized calcium (iCa) (**D**). (**E**) Representative images of H&E-stained images of pancreatic sections from rats infused with GTO alone or GTO plus PPL. Scale bars: 200 μm. (**F**–**H**) Plots of preterminal oxygen saturation (Ox sat) (**F**), LDH activity in BAL fluid from the lungs (**G**), and protein concentrations in the BAL (**H**). (**I**) Representative lung histologic images after H&E staining from each group mentioned at the top of the image. Scale bars: 100 μm. The rectangle inset is zoomed outside the ×20 image. In the PPL plus GTO group, arrows point to alveolar wall damage, and asterisks show fluid-filled alveoli. All data in graphs were compared by ordinary 1-way ANOVA with multiple comparisons.

To determine the role of lipolytic NEFA generation on HTG-AP severity, we studied whether organ failure, which defines pancreatitis severity in humans (30), could be induced in experimental models by intravascular lipolysis during hypertriglyceridemia. For this we first induced HTG (Figure 2, A–G) in the UFA-fed C57bl/6 mice (WT) by administering poloxamer-407 to these mice on day –1 (as described in Methods). We then induced pancreatitis (HTG + AP) using IL-12 and IL-18 in some of these (red symbols in Figure 2, A–G), whereas others were followed with only HTG (blue symbols). We also induced similar HTG-AP mice in UFA-fed mice that had genetic deletion of the pancreatic TG lipase (*PNLIP*) gene, herein referred to as PTL-KO mice (green symbols in Figure 2, A–G). Mice with HTG alone (blue symbols in Figure 2, A–G) were used as controls. Mice in all groups had increased serum TG levels from less than 100 mg/dL at baseline to the 15,000–25,000 mg/dL range within 1 day of HTG induction. Serum TG levels were similar in all groups before AP induction (day 0 in Figure 2B). These were 18,667 ± 2,402 mg/dL in HTG-only controls, 21,588 ± 4,245 mg/dL in C57bl/6 mice before HTG-AP, and 19,597 ± 4,450 om PTL-KO mice (*P* = 0.83). Mice with AP (induced on day 0 after blood collection) had a significant increase in serum lipase by day 1 (Figure 2, A and B). AP caused a rapid reduction in serum TGs (red dots in pink oval, Figure 2B) but not in mice with HTG alone (blue, Figure 2B) or in PTL-KO mice (green dots, Figure 2B). The reduction in TGs resulted in a corresponding significant increase in serum NEFAs (red dots in pink oval, Figure 2C) on day 1 of AP. Mice with HTG alone had no change in serum TGs or NEFAs on day 1 (blue dots in blue ovals in Figure 2, B and C). Similarly, PTL-KO mice had no change in serum NEFAs on day 1 of AP (green dots Figure 2C). Between day 1 and 2, WT mice with AP began to appear moribund and required euthanasia. WT mice with HTG-AP had higher serum creatinine levels at euthanasia (Figure 3D) and a preterminal reduction in pulse distention consistent with hypotension (19, 31) (Figure 3E). WT mice also developed generalized hypothermia (Figure 3F), along with an 80% reduction in survival by day 3 (Figure 3G). These findings are consistent with HTG-AP induction of multisystem organ failure. WT mice had 4.7% ± 5.3% pancreatic parenchymal necrosis, seen as pale pink areas with loss of cellular detail (arrows in Figure 2H). This necrosis was reduced to 0.6% ± 0.7% (*P* < 0.01) in PTL-KO mice (Figure 2, I and J). The pancreatic edema score averaged 2/4 in both groups of mice (Figure 2K).

To determine the specific roles of unsaturated NEFAs, we compared organ failure parameters in mice given the saturated NEFA palmitic acid versus the unsaturated NEFA linoleic acid. Both of these NEFAs were increased in patients with HTG-AP (Table 1). Only linoleic acid increased blood urea nitrogen (BUN) levels and lowered carotid pulse distention, which indicated hypotension (19, 31), worsened hypothermia (Figure 2, L–N), and increased apoptotic cells in the lungs, consistent with acute lung injury (Supplemental Figure 1; supplemental material available online with this article; https://doi.org/10.1172/JCI184785DS1), resulting in a moribund appearance of the mice, requiring euthanasia by day 3. Therefore, lipolytic release of unsaturated NEFAs like linoleic acid may worsen AP outcomes.

Severe AP includes respiratory and renal failure. We thus studied whether intravascular lipolysis of TGs worsens HTG-AP in a distinct and direct intravascular lipolysis model of HTG in rats. For this, we intravenously infused TGs (glyceryl trioleate [GTO], TGs composed of 3 oleic acid chains) into precannulated rats, alone or with PPL and the lipase inhibitor orlistat. GTO infusion increased serum TGs from 94 ± 29 mg/dL to 233 ± 120 mg/dL (*P* = 0.0004; Supplemental Figure 2A). As shown in Figure 3, PPL infusion increased lipase (Figure 3A), which was inhibited by orlistat. As shown in Figure 3B, the PPL + GTO group had the highest NEFA levels, consistent with lipolysis of TGs, which was prevented in the PPL + GTO + orlistat group. The PPL + GTO group also had significantly higher serum BUN levels (Figure 3C) and lower ionized calcium levels (Figure 3D), which were prevented by orlistat. PPL + GTO treatment only induced edema in the pancreas but no necrosis or inflammation, whereas levels in other groups remained similar to those in controls (Figure 3E and Supplemental Figure 2B). While infusion of PPL or GTO did not cause any changes in oxygen saturation, infusion of PPL + GTO together caused a significant decrease in the percentage of oxygen saturation (Figure 3F) compared with baseline, requiring euthanasia. This was associated with an increase in lactate dehydrogenase (LDH) levels and protein concentrations in the bronchoalveolar lavage (BAL) fluid, supporting lung injury (Figure 3, G and H). Histologically the PPL + GTO group had fluid in the alveoli that was seen as diffuse pink staining (asterisks in Figure 3I), along with damage to the alveolar walls (arrows in Figure 3I, PPL + GTO panel). However, infusing orlistat in addition of PPL and GTO (PPL + GTO + orlistat) prevented the drop in oxygen saturation, the LDH and protein increase, and histological changes noted in the PPL + GTO group. Thus, intravascular lipolysis of circulating TGs can cause lung injury and organ failure with minimal pancreatic injury during severe AP.

### The correlation of serum lipase with NEFAs increases by factoring in the corresponding TGFAs.

**Figure 4:**
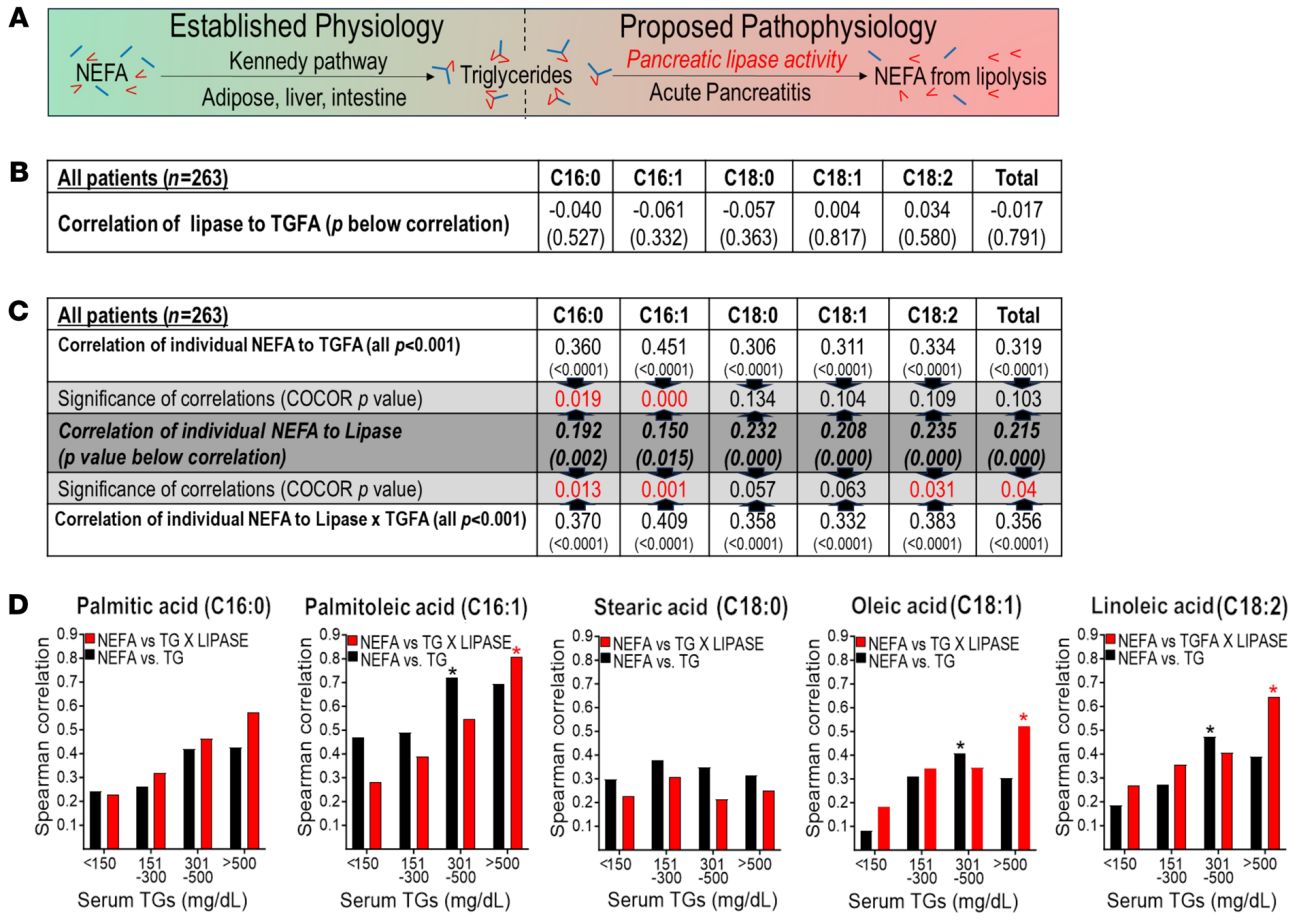
Effect of lipase activity on the relationship between individual NEFAs and TGFAs in patients with pancreatitis. (**A**) Schematic comparing the Kennedy pathway (left side, green background) by which NEFAs are physiologically incorporated into the TGs for storage (adipose, liver) or transport (intestine) versus the pathological release of NEFAs from intravascular TG lipolysis by pancreatic lipases during HTG-AP (red background on the right side). (**B**) Correlation of serum lipase activity with individual TGFAs for all patients. Each column shows a unique FA. The upper value shows the correlation coefficient, and the lower number the *P* value. (**C**) All patient data were formatted as in **B**. Middle row (dark gray background) correlates individual serum NEFAs with serum lipase; top row (white background) correlates individual NEFAs and their TGFA concentrations; and bottom row (white background) correlates individual NEFAs and the product of serum lipase × TGFA concentrations. The light gray rows show *P* values comparing the strength of correlations (COCOR as described in Methods) between the middle row and corresponding top or bottom rows. Those with a *P* value of less than 0.05 are shown in red. (**D**) Bar graphs of correlations (*R* values) arranged by serum TG concentrations (*x* axis) for individual FAs. Each graph is for a FA (mentioned above) and shows correlations of its NEFAs with corresponding TGFAs (back bars) or NEFAs with the product of the corresponding TGFA concentration × serum lipase (red bars). Asterisks show the bar with significantly stronger correlations versus normal TGs, i.e., TGs below 150 mg/dL. All correlations are Spearman correlations, and *P* values are 2 tailed. The comparison of COCOR correlations between 2 Spearman coefficients were done as described in Methods, and *P* values are shown. Asterisks in **D** indicate a COCOR *P* value of less than 0.05 versus the normal (<150 mg/dL) TG group.

We next aimed to understand the relation between circulating NEFAs and TGs, i.e., whether TG lipolysis leads to NEFA formation in AP or if the FAs in TGs (i.e., TGFAs) merely correlate with their precursor NEFAs. For this, we first correlated specific NEFAs to their TGFA concentrations for all patients, which would support synthesis via the Kennedy pathway (Figure 4A). To determine whether TGFA hydrolysis by lipase generates NEFAs, as would happen during AP, we factored in serum lipase levels while correlating individual NEFAs to the corresponding TGFAs, i.e., we correlated NEFAs to TGFA × lipase. We also correlated NEFA concentrations to the corresponding serum lipase activity for all patients.

On initial analysis, abnormal distributions were noted for individual NEFAs (Supplemental Figure 3), and also for serum lipase (i.e., enzyme), TGFAs (the substrate), and the multiplicative product of (TGFA × lipase) (Supplemental Figure 4). While serum lipase did not correlate (Spearman) with any TGFA concentration (Figure 4B), serum lipase in AP did have a weak but significant Spearman correlation with each of the major NEFAs measured (dark gray, middle row, Figure 4C). We then correlated the concentrations of specific NEFAs to their corresponding TGFAs for all patients with AP. NEFA concentrations of palmitic acid (PA) (C16:0) and palmitoleic acid (POA) (C16:1), correlated more strongly with corresponding TGFAs (upper row, Figure 4C) than with serum lipase. In this NEFA-versus-TGFA correlation for PA, POA was not improved by multiplying the TGFA concentrations by lipase (bottom row, Figure 4C), despite the latter remaining stronger than the correlation with lipase. Therefore, PA and POA in the NEFA and TGFA fractions correlated independently of lipolysis, consistent with the Kennedy pathway contributing to their relationship in all patients with AP. However, for total NEFAs and, specifically, for linoleic acid (C18:2) NEFAs, the correlation improved significantly after multiplying their TGFA concentrations by serum lipase (*P* = 0.03, bottom row in Figure 4C).

To determine the relevance of these correlations to HTG-AP, we stratified the correlations by serum TG concentrations in mg/dL (Figure 4D). For this, serum TG levels below 150 (*n* = 107; or controls), 151–300 (*n* = 101), or 301–500 (*n* = 35), and above 500 mg/dL (*n* = 27) were arranged on the *x* axis with Spearman correlations on the *y* axis. The mean, median, and other descriptors of these groups are shown in Supplemental Figure 5. The black bars in Figure 4D depict NEFA-to-TGFA correlations (relevant to the Kennedy pathway). The red bars show NEFA correlations to lipase × TGFAs (supporting lipolysis). While NEFA-to-TGFA correlations of unsaturated palmitoleate, oleate, and linoleate increased up to TGs below 500 mg/dL (black asterisk in Figure 4D), these correlations weakened at TG values above 500 mg/dL. Interestingly, at TG levels above 500 mg/dL, factoring in the lipase activity (red bars) further strengthened the NEFA-to-lipase × TGFA correlations of POA (*r* = 0.8), linoleic acid (LA) (*r* = 0.62), and oleic acid (OA) (*r* = 0.52) versus corresponding correlations at TGs below 150 mg/dL (all with *P* < 0.05, red asterisk). Therefore, during AP, lipolysis of linoleate, palmitoleate, and oleate from their TGs increased with lipase activity at TGs of greater than 500 mg/dL. The weaker TGFA-NEFA correlations at TGs above 500 mg/dL support this. Interestingly, neither correlation increased with TG concentrations for the saturated FAs stearic acid (SA) and PA. These findings supporting lipolytic generation of unsaturated NEFAs from TGs during AP and the established injurious roles of lipolytically generated unsaturated NEFAs (21, 24, 32) are consistent with HTG-AP being more severe (2, 7, 8, 14, 33–35).

We also correlated the proportions of individual NEFAs versus TGFAs in relation to serum TG levels, double-bond number (i.e., unsaturation), and FA chain length (Supplemental Figure 6). When comparing the correlations (COCOR) among FAs based on the double-bond number using R (α 0.05; CI, 0.95) (36, 37), we noted Pearson coefficients to be significantly higher for linoleic acid than oleic acid or stearic acid (which have 18 carbon atoms; *P* < 0.001). Among 16 carbon FAs, COCOR for POA was stronger than palmitic acid (*P* < 0.001; Supplemental Figure 6, A–F). These findings are consistent with those of previous studies showing that unsaturation improves the fit of TGs in pancreatic lipases (23). All FAs except stearic acid had stronger correlations at TGs above 200 mg/dL (*n* = 111 patients, Supplemental Figure 6, G–L). Equivalent results were obtained when the cutoff was greater than 150 mg/dL (*n* = 162, data not shown). Overall, these findings agreed with breakdown of TGFAs contributing to the unsaturated NEFAs increased during HTG-AP.

Last, to experimentally verify the clinical observations, we used TGs containing linoelic acid, oleic acid, palmitic acid, and palmitoleic acid in various combinations and exposed them to lipases released by acinar cells in suspension. As shown in Supplemental Figure 7A, lipolysis of the TGs of palmitic acid (C16:0), i.e., tripalmitin (PPP) was negligible and increased significantly by replacing 2 acyl chains in the TGs with POA (C16:1; PO) in PO(2)-P. Similarly, addition of the saturated palmitate (C16:0) to the TGs of linoleate or oleate (i.e., using 1,2-dilinoleoyl-3-palmitoyl-rac-glycerol [LLP] or 1,2-dioleoyl-3-palmitoyl-rac-glycerol [OOP] in place of glyceryl trilinolein (LLL) or triolein (OOO) also reduced lipolysis of their TGs by 50%–80% (Supplemental Figure 7B). Moreover, replacement of 1 linoleic acid in LLP with an oleic acid, i.e., LOP, further reduced TG lipolysis by more than 90%. Therefore, while linoleic acid (C18:2) had the highest fidelity of lipolysis by pancreatic lipases, adding oleic acid (C18:1) and palmitic acid (C16:0) incrementally decreased this fidelity. These results support the concept that double bonds (i.e., unsaturation) in long-chain FAs increased their lipolysis from a TG and that saturation interfered with TG lipolysis (23). Overall, our results show that excessive generation of unsaturated NEFAs from circulating TG lipolysis may have caused organ failure and worsened HTG-AP severity.

## Discussion

**Figure 5:**
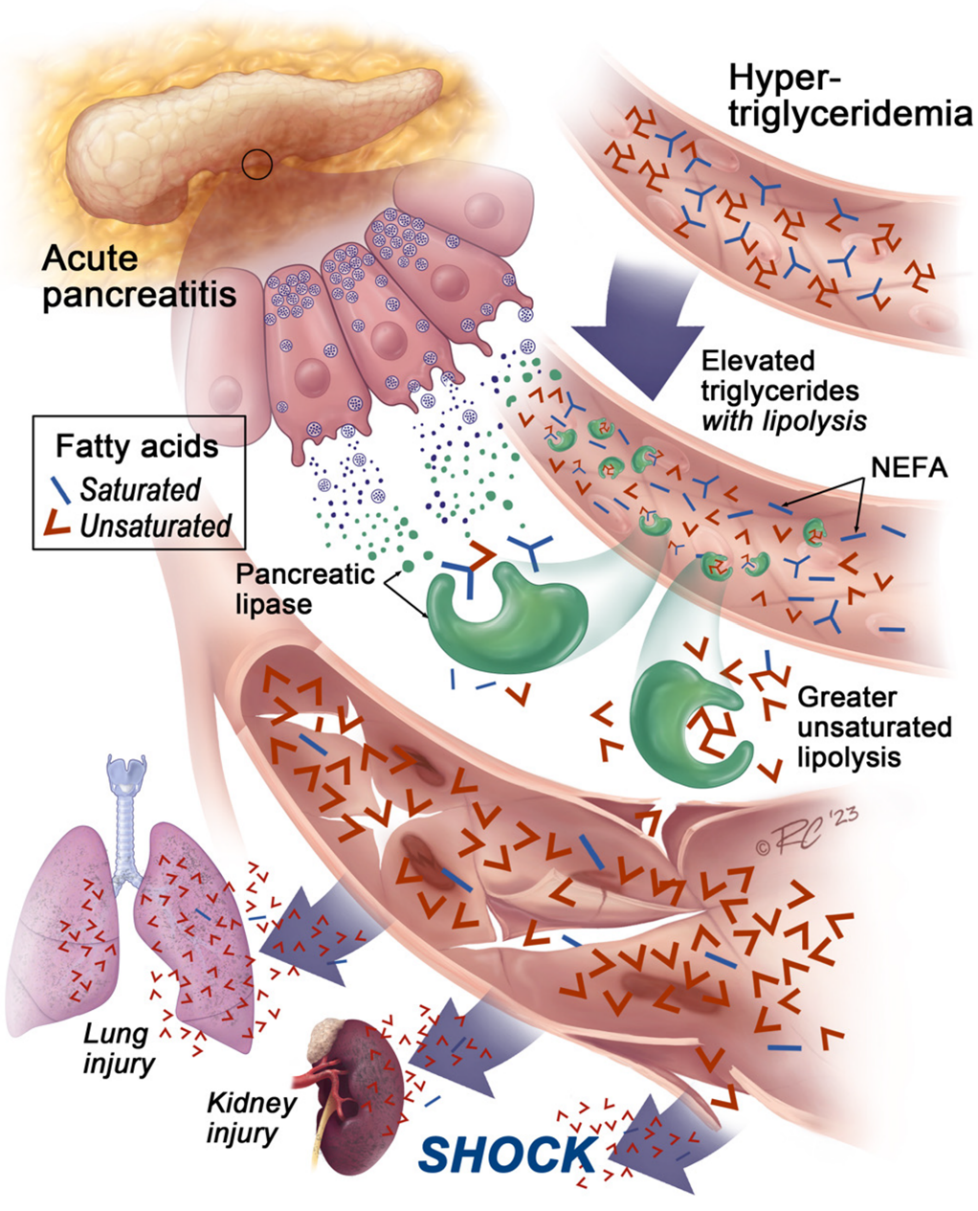
Schematic showing mechanisms underlying severity of HTG-AP. Illustration shows elevated TGs (3-limb structures in blue, red shown is within upper blood vessels) being cleaved by pancreatic lipases (green) into FAs during HTG-AP (middle vessel). The images, inset depict saturated FAs as blue lines, and unsaturated FAs as red. The unsaturated FAs cause vascular leakage, resulting in shock along with renal failure and lung injury, resulting in severe AP (lower vessel).

Here, we examined the reasons underlying the greater severity reported in patients with HTG-AP (2, 7, 8). These patients had higher serum NEFA levels (Table 1) that caused organ failure and severe AP (Figures 2 and 3). The role of NEFAs in worsening clinical AP is well known (16–18) and is supported by numerous mechanistic studies (19, 21, 22, 38, 39). Here, we noted that during HTG-AP, serum NEFAs had a stronger correlation with the product of multiplying unsaturated TGFA concentrations with serum lipase activity than with either lipase activity or TGFAs alone (Figure 4D). Figure 5 summarizes these findings. It shows that lipolysis during HTG-AP preferentially generates unsaturated NEFAs, which injure cells and cause organ failure as previously shown (19, 22, 23). These findings also agree with previous studies showing that unsaturation increases TG hydrolysis to NEFAs by the pancreatic lipases released in AP (23) and that HTG-AP is detrimental, irrespective of AP etiology (2, 7, 8). Overall, this study shows that worse outcomes in HTG-AP were due to elevated NEFAs generated from intravascular lipolysis of circulating TGs.

The study highlights the implications of using heparin, which releases lipoprotein lipase and may generate NEFAs when used alone or during plasmapheresis to treat HTG-AP, since several studies showed no improvement in outcomes in such scenarios despite a reduction in serum TGs (40–43). The study also explains the worse outcomes reported in patients with pancreatitis receiving parenteral nutrition (44–46), since intravenous lipid emulsions contain TGs that can be hydrolyzed to NEFAs by the circulating lipases. It also brings forth the relevance of a lipase inhibitor (RABI-767) in phase II clinical trials (NCT06080789) for preventing severe AP.

No association between severe AP and hyperviscosity syndromes (14) has been reported. Moreover, patients with lipoprotein lipase deficiency (median serum TGs of 29 mM or ≈2,500 mg/dL) experience 1 AP episode every 3–5 years (47) and have a lifetime risk of ≈35% (48). Therefore, hyperviscosity or HTG alone does not completely explain the risk of severe AP. These observations and the current study support an alternate mechanism, i.e., that intravascular TG lipolysis leads to severity. Previous studies have associated diabetes with the risk of developing HTG-AP (49, 50). While both obesity (51, 52) and HTG (18, 53, 54) are risk factors for severe AP, the role of diabetes in severe outcomes remains unclear (55, 56). We cannot comment on the role of alcohol in HTG-AP in the current study, since the non–HTG AP group included patients with alcoholic AP (22%) based on clinical history. Moreover, the history-based distinction between “alcohol use disorder with acute pancreatitis” versus “alcohol-associated pancreatitis” is hard to validate, since current guidelines (57) use the history-based consensus statement of the Zurich Workshop of 1996 (58). Moreover, patients often under-report alcohol intake (59). Whether objective markers like serum FA ethyl esters will improve diagnosis of alcoholic AP remains to be seen (27).

We note the serum (pancreatic) lipase activity strengthened the correlation of NEFAs with TGFAs (Figure 4), which was most during HTG AP (red bars in Figure 4D). Moreover, the 8- to 10-fold higher molar TG (i.e., substrate) concentrations in the sera of patients with HTG-AP (561–1,064 mg/dL; i.e., 8.5 mM [6.6–12.5 mM]) versus NEFAs (0.95 [0.6–1.5 mM]) (Table 1) make the lipolytic generation of NEFAs (i.e., product) during early AP a very plausible argument. Thus, we mechanistically sought to determine whether intravascular breakdown of TGs to NEFAs can cause the organ failure previously attributed to NEFAs (19, 21, 23, 24, 32).

Our experimental studies involved 2 distinct models using mice and rats. TGs peaked at 15,000–25,000 mg/dL (150–300 mM) in mice (Figure 2B), whereas in rats, GTO infusion increased serum TGs only to the 150–400 mg/dL (2–5 mM) range (Supplemental Figure 2A). Despite these differences in TG levels, lipolysis of TGs in both models resulted in organ failure, irrespective of whether it was by intravenous lipase or interleukin-induced pancreatitis. Moreover, the organ failure was independent of the extent of pancreatic necrosis. The high serum TGs in the group that had HTG alone (blue dots in Figure 2B) normalized slowly and uneventfully over 6–7 days by normal clearance mechanisms. These experimental studies showed organ failure results from a rapid NEFA increase due to circulating lipase hydrolyzing the HTG during IL-12– and IL-18–induced AP (red symbols in Figure 2, A–G) or intravenous lipase infusion or (Figure 3), and not by HTG alone or only elevated lipase. These are consistent with previous studies showing that a rapid increase in NEFAs, which exceeds the binding capacity of their carrier albumin, results in higher unbound FAs that eventually mediate the cellular events culminating in organ failure (21, 23, 32). This organ failure is noted as renal injury, lung injury, and hypoxia, along with hypotension resulting in reduced survival. Genetic deletion of pancreatic TG lipase (green dots in Figure 2, A–G) or intravenous coadministration of the lipase inhibitor orlistat (Figure 3) prevented this organ failure and improved survival by preventing hydrolysis of circulating TGs to NEFAs. These data strongly support the clinical findings that persistent organ failure, which defines severe HTG-AP is due to lipolysis of the circulating TGs to NEFAs. We also note that unsaturated NEFAs like linoleic acid, which are more hydrolyzed in HTG-AP (Figure 4D), also cause organ failure in mice (Figure 3, H–J and Supplemental Figure 1), unlike palmitic acid, which is less hydrolyzed and is also less aqueously stable (23) .

The above findings are also consistent with our data (Supplemental Figure 7) and previous studies showing that fewer double bonds or saturated FAs in a TG molecule reduce its lipolysis by pancreatic lipases (23). For example, replacing linoleic acids (C18:2) in the TG LLL to LLP and LOP reduced their glide scores for active human pancreatic TG lipase from –7.16 to –4.72, and –1.334, respectively (23). This also increased the distance between the ester linkage in TGs and the lipases’ catalytic serine that hydrolyzes the ester linkage from 4.04 A° to 9.99 A° and 12.42 A°, respectively (23). These published data are in concordance with our current finding of a correlation of LA as NEFAs, with corresponding TGFAs being stronger in HTG-AP than saturated TGs (Figure 4D).

The high TG levels we observed in mice given poloxamer-407 is a potential limitation of our studies. However, serum TGs of 100–200 mM have also been noted in humans (60) and in HTG-AP (12, 13). The WT-HTG mice without HTG-AP (blue dots in Figure 2, A–F) had uneventful normalization of TGs over a 1-week period. In rats, GTO (stock 100 mM) was infused intravenously at approximately 5% blood volume/hour (i.e., 1 mL/hour), thus delivering a blood concentration of 5 mM/hour. However, this only achieved a 2–5 mM concentration over 8 hours (Supplemental Figure 2A). These data indicate rapid physiologic TG clearance and were considered while designing the current experiments, although the relevance of the high serum TGs in mice remains to be determined.

To summarize, the study shows that the increased severity noted during HTG-AP may be due to the increased serum NEFAs generated as a result of the lipolysis of elevated circulating TGs during pancreatitis. Whether prevention of this lipolysis with agents in ongoing clinical trials (NCT06080789) will improve outcomes compared with modalities using heparin (40), which reduces TGs by releasing lipoprotein lipase (61) and causing lipolysis, remains to be seen.

## Methods

### Sex as a biological variable

All data were accrued chronologically over the period of the study, and no alterations were made on the basis of the patient’s sex. Differences in outcomes were analyzed on the basis of sex and are reported in Results. All mice that reached 20% or higher body fat by weight on the high-fat diet were used in the study. These included both females and males and had similar outcomes.

### Human serum processing

Patients presenting to the Mayo Clinic Arizona ER between August 2019 and November 2021 with lipase levels more than 3 times the ULN were automatically flagged by the system. The initial storage, transportation (4°C), aliquot, and final storage (–80°C) of the residual serum samples collected at admission were done as described previously (21). The transportation and storage time (i.e., hours to processing) were recorded for each sample. All analyses were done at the time of the first thaw from –80°C. A colorimetric TG assay was done using Thermo Fisher Scientific reagent as described in the manufacturer’s protocol. For FA composition of TGs and NEFAs, the serum samples stored at –80°C were shipped on dry ice to the Vanderbilt University lipidomic core and analyzed by gas chromatography as described previously (19). The analysts were blinded to the nature of the human serum samples. Total oleic acid (O; C18:1) was calculated by adding C18:1ω9 and C18:1ω7.

### Patient data collection

Patient details including demographics, diagnosis, etiology of pancreatitis, serum lipase levels, and clinical outcomes including length of stay, ICU admission, and presence and duration of organ failure were determined according to the chart review and notes by the treating physicians. Diagnosis of AP was established if the patient met at least 2 of the 3 diagnostic criteria (30), as recommended. Patients with TGs of greater than 500 mg/dL were diagnosed with HTG-AP according to the AHA 2018 guidelines (29), and all other patients were classified as non–HTG-AP. Organ failure in patients during hospitalization was determined on the basis of a modified Marshall score of greater than 2 (30). The presence of organ failure for more than 48 hours was considered to be persistent organ failure and required for a diagnosis of severe AP (30). We compared demographics including age, sex, race, BMI, diabetes, AP etiology, admission serum lipase levels, TGs, NEFAs, ICU admission rates, and organ failure rates among HTG and non–HTG-AP patients.

### Quality control of serum samples

The effect of storage and transportation (time) on artifactually increasing NEFAs (which are one-tenth the molar concentration of TGs; shown later in study) from TGs was accounted for using 2 methods. (a) Correlation of NEFA concentrations with the duration of storage: the normality of distribution was first determined for each parameter and noted to be abnormal for each (Supplemental Figure 3). The sample’s NEFA concentrations (total and individual NEFAs) were then Spearman correlated with their respective storage duration (in hours). As seen in Supplemental Figure 8, there was no correlation between the duration of storage and NEFA concentrations. (b) Measurement of NEFA generation under storage conditions after artificially increasing serum TGs: baseline serum NEFAs were measured. Ten millimolars of the mixed TGs (composition shown below) was then added to the patients’ sera (*n* = 10, with serum lipase 1,668 ± 902 U/L). NEFA generation was measured daily over 3 days under conditions in which the samples were stored and transported (i.e., 4°C). The TG proportions (in brackets) added were (OOP[6], LOP[6], PLP[2], OPO[4], LLP[1], PO(2)-P[1])X. The resulting percentage of TGFAs in this mixture represent middle quartiles of our patients, with oleic acid at 43.3%, palmitic acid at 33.3%, linoelic acid at 20%, and POA at 3.3%. As shown in Supplemental Figure 9, there was no significant change in serum NEFAs noted on ANOVA compared with baseline whether these were compared in absolute concentrations (Supplemental Figure 9A) or as a change from baseline NEFAs taken as 100% (Supplemental Figure 9B.)

### Reagents

LLL, OOO, tripalmitin (PPP), and mixed TGs LLP, 1-palmitoyl-2-oleoyl-3-linoleoyl-rac-glycerol (LOP), OOP, linoleic acid (L), palmitic acid (P), oleic acid (O), DMSO, and porcine pancreatic lipase (PPL) were purchased from MilliporeSigma. 1,2-dipalmitoleoyl-3-palmitoyl-*rac*-glycerol [POA(2)-P] was purchased from Cayman Chemical. All TGs were sonicated as 20X stocks in PBS pH 7.4 before use. Tripalmitin, which is a solid powder and cannot be sonicated, was first dissolved in DMSO as a 60 mM stock and diluted 200 times (0.5% DMSO) before use. Poloxamer-407 (MilliporeSigma) was freshly dissolved in sterile PBS at 80 mg/mL before use. NEFAs in cell media (discussed below) were measured using the LabAssay NEFA kit from Fujifilm (Wako Chemicals). Lipase assays for cellular studies (discussed below) were conducted using the manufacturer’s protocol as previously described (23). The lipase assay for human serum samples was performed using COBAS (Roche Diagnostics) according to the manufacturer’s protocol.

### Animal studies

#### Experimental pancreatitis.

CD1 mice (Charles River Laboratories) or C57BL/6J mice (The Jackson Laboratory) were acclimatized for at least 2 days before experimentation. Mice were housed at temperatures ranging from 21°C–25°C under a 12-hour light/12-hour dark cycle and allowed to drink ad libitum. There were 13–14 mice in each group.

#### HTG in mice.

Male and female C57BL/6J mice (18–26 weeks old) were used after feeding an unsaturated high-fat diet from age 6 weeks onwards, as previously described (23). HTG was induced by a single 1 g/kg dose of poloxamer-407 intraperitoneally on day –1, as described previously (62). Poloxamer-407 or pluronic F127 is a nonionic detergent (63) commonly used to induce HTG in mice (62). This dose was based on standardization to achieve stable HTG for 48 hours while avoiding repeated dosing. There were no adverse effects noted in such mice, which were followed for up to 1 month.

#### HTG-AP in mice.

The day after administering poloxamer, AP was induced by administration of the first dose of IL-12 and IL-18 (i.e., day 0) as described previously (23, 64). For this, murine recombinant IL-12 (Peprotech, 150 ng/30g) and IL-18 (R&D Systems, 750 ng/30 g) were used. A second dose of IL-12 and IL-18 was repeated a day later as per established protocol (64). Daily monitoring included general activity and carotid pulse distension (MouseOx Oximeter, STARR Life Sciences) as a noninvasive measure for blood pressure (31, 65) and blood flow in conscious mice and rectal temperatures. Mice were euthanized using carbon dioxide if they appeared moribund or after 5 days, which ever came first. Blood was processed for biochemical assays (serum) as described below and previously (19, 38).

#### In vivo NEFA studies in mice and rats.

To minimize strain-specific effects, 10- to 12-week-old male CD-1 mice were used for this experiment (*n* = 6/group) as part of a study described previously (23). The different groups included control mice or mice treated with palmitic acid (0.3% body weight) or linoleic acid (0.2% body weight). These mice were followed and euthanized as described above. Blood and histology studies were done as described previously (19, 22, 38).

### Intravascular TG lipolysis in rats: infusion in rats

Precannulated Wistar rats (Charles River Laboratories) were used for the experiments. There were 4 or more rats in each group, which had an indwelling jugular venous catheter. Maintenance of patency was done according to the instructions from the supplier. TG suspensions of sterile 10% glyceryl trioleate (GTO) in sterile lactated ringers were made alone or with orlistat (50 mg/mL GTO). PPL stock (100 mg/mL) was dissolved in sterile lactated Ringers and syringe filtered. This stock was used as 1:19 in the GTO suspension (as PPL + GTO or PPL + GTO + orlistat). Infusions were done at the rate of 18 μL/min for 8 hours or until the rats met euthanasia endpoints (e.g., moribund, tachypneic, or in distress), whichever came first. Monitoring included general activity, heart rate, and carotid pulse distension (MouseOx Oximeter, STARR Life Sciences) as a noninvasive measure for blood pressure (31, 65) and oxygen saturation (MouseOx Oximeter, STARR Life Sciences) as a measure of pulmonary function. Rats were euthanized using carbon dioxide if they appeared moribund or after 8 hours, which ever came first. Blood was processed for biochemical assays (serum) as described below and previously (19, 38). Preterminal BUN was measured using iSTAT as a marker of renal function. BAL and histology studies were done on H&E-stained formalin-fixed, paraffin-embedded sections as previously described (22).

### Cell studies

Pancreatic acini were harvested from CD-1 mice in HEPES buffer as previously described, and viability was confirmed to be greater than 95% (19, 23). All TGs were used at a final concentration of 300 mM (0.3 mM) in the cellular studies. Hydrolysis of TGs was measured as the increase in NEFA concentrations in the medium over 15 minutes.

### Statistics

After blinded quantification of patients’ lipid parameters, the serum TG and NEFA concentrations for palmitic, POA, stearic, oleic d, and linoleic acids were entered in separate columns of a master Excel file with 1 row for each patient. Other variables including demographics and AP parameters were retrieved from chart review. Categorical data for both mice and human studies were compared using the χ^2^ test. The normality of continuous sample distributions was determined, and outliers were identified using the ROUT method with a *Q* value of 1%. Continuous variables were compared using a Mann-Whitney *U* test. A *P* value of 0.05 or less was considered significant. To determine correlations between proportions (Supplemental Figure 6), Pearson correlations (*r*) were measured between proportions of FAs in TGs and the corresponding FAs in NEFAs after excluding outliers. Strengths of correlations in Figure 4 were compared using the comparison of correlations (COCOR) package in R (36, 37). Additionally, differences in Pearson correlations of samples with TGs below 200 mg/dL versus TGs above 200 mg/dL deduced using COCOR were confirmed at Online-Calculator for testing correlations and Psychometrica using the comparison of correlations from independent samples. The analyses were conducted in R 4.3.0 (R Foundation for Statistical Computing), and all graphs were constructed using GraphPad Prism, version 9 (GraphPad Software). Continuous data are mentioned in text as the median and IQR and represented graphically as boxes ranging between the IQR and whiskers extending from minimum to maximum levels. Categorical data are presented as percentages and depicted as bar graphs.

### Study approval

All human studies were approved by the Institutional Review Board of Mayo Clinic. All experiments were approved by the IACUC of the Mayo Clinic. Animals were handled in accord with the *Guide for the Care and Use of Laboratory Animals* (National Academies Press, 2011) by the Institute for Laboratory Animal Research.

### Data availability

Supporting data and values can be made available from the corresponding author upon reasonable request. Values for all data points in graphs are reported in the Supporting Data Values file.

## Author contributions

Acquisition of data was facilitated and carried out by PR, BK, MS, SK, AA, ME, MM, ANP, ST, BM, and SJ. Analysis and interpretation were done by PR, ST, BK, BM, SK, MS, MM, YHC, and VPS, who also helped in the critical evaluation of the manuscript. The manuscript was drafted by PR and VPS. Statistical analysis was done by PR, YHC, and VPS. VPS and CS supervised the study, and VPS designed and conceptualized the study.

## Supplementary Material

Supplemental data

ICMJE disclosure forms

Supporting data values

## Figures and Tables

**Table 1:**
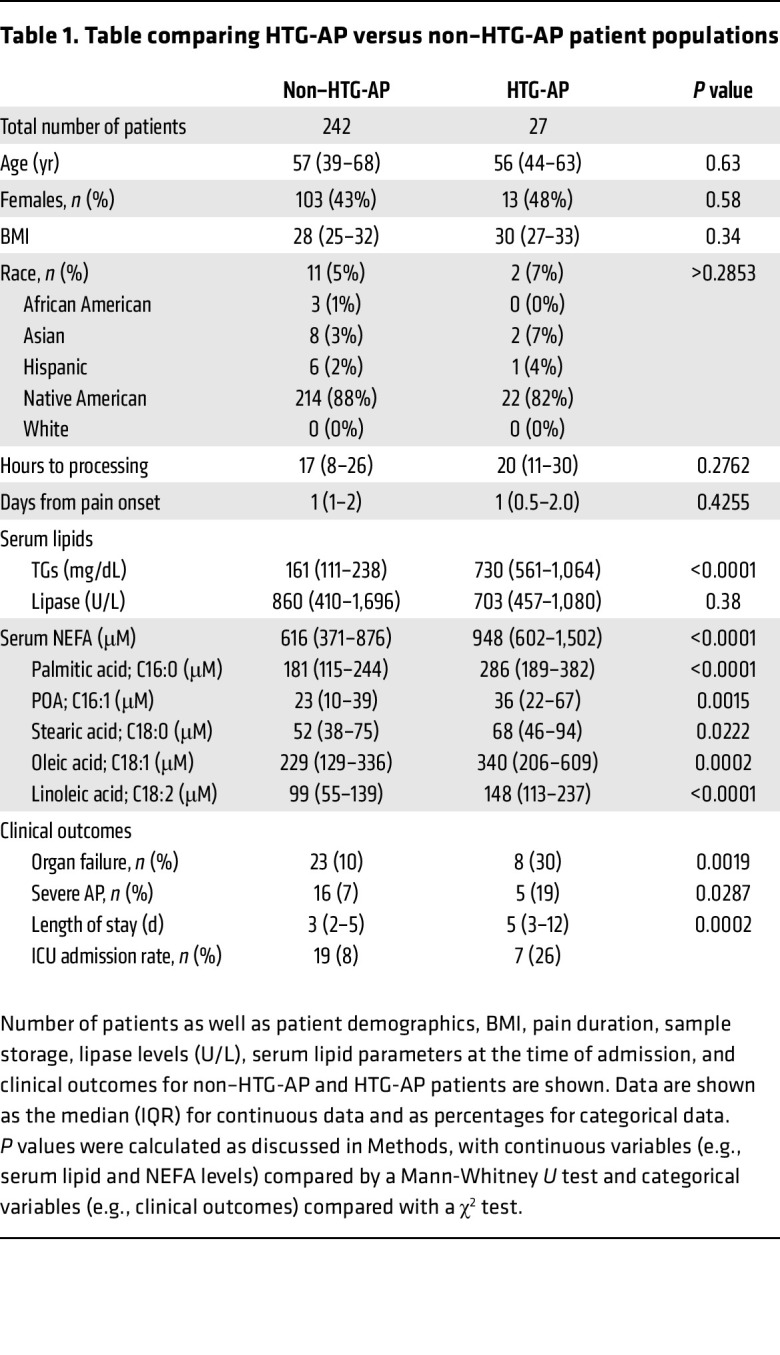
Table comparing HTG-AP versus non–HTG-AP patient populations

## Version 1 11/07/2024

In-Press Preview

## Version 2 01/02/2025

Electronic publication
