# Supplementary material for: Prospective observational study and mechanistic evidence showing lipolysis of circulating triglycerides worsens hypertriglyceridemic acute pancreatitis
Source: J Clin Invest. 2024 Nov 7;135(1):e184785. doi: 10.1172/JCI184785 (PMC11684810; doi:10.1172/JCI184785)
Supplement: Supplemental data [file jci-135-184785-s093.pdf]

**Supplementary Data:**

# Supplementary Figure -1

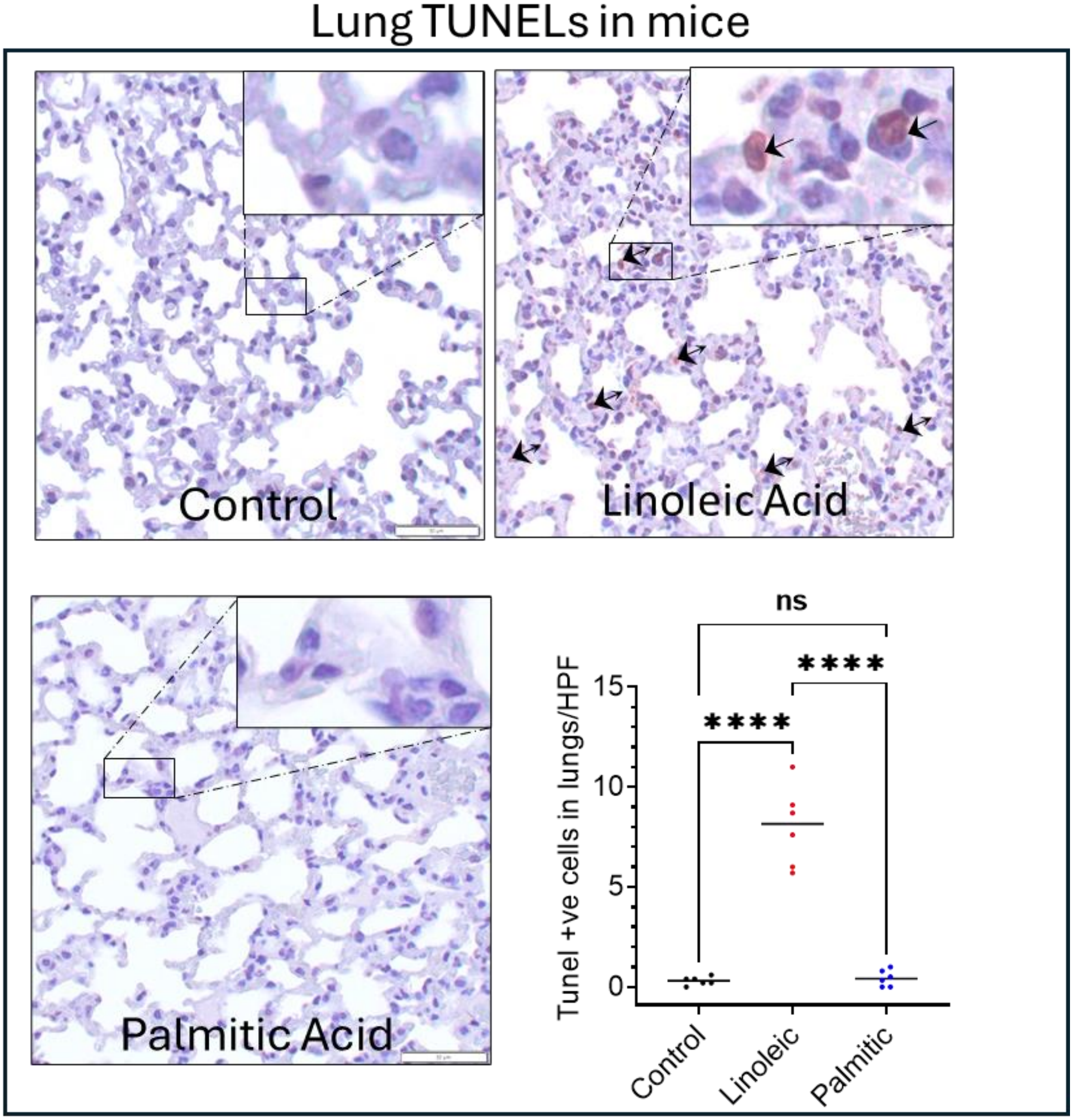


**Supplementary figure 1:** TUNEL staining of lungs of mice given Linoleic acid, palmitic acid vs. controls. The representative images for each treatment are shown above their respective labels and the graph shows mean with a dot representing each mouse. Asterisks show significance by an ordinary one-way ANOVA.

**Supplementary figure -2**

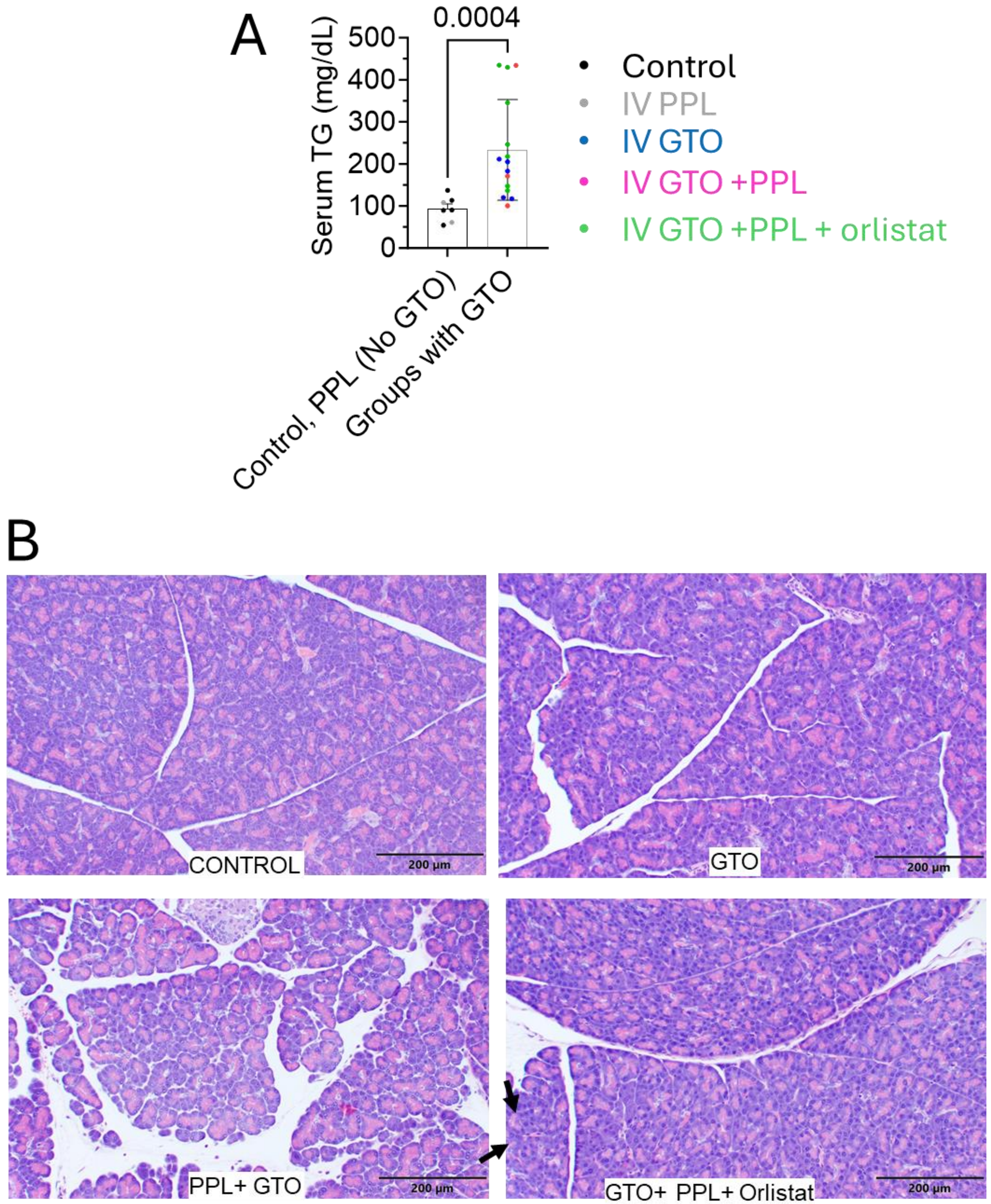


<u>**Supplementary figure 2:**</u> **A:** Bar graphs with color coded individual values and standard deviation comparing the serum triglycerides in the corresponding groups. The p-value shown in by a Mann-Whitney test. **B:** Representative images of Hematoxylin and eosin-stained pancreatic sections from rats from each group mentioned below the image.

# Supplementary figure -3

## Normality of distribution

| | Time | 16:0 | 16:1 | 18:0 | 18:1w9 | 18:1w7 | total C18:1 | 18:2 | total NEFA |
|---|---|---|---|---|---|---|---|---|---|
| **Test for normal distribution** | | | | | | | | | |
| **Anderson-Darling test** | | | | | | | | | |
| A2* | 14.78 | 6.178 | 15.19 | 11.43 | 6.815 | 12.37 | 7.052 | 6.834 | 6.547 |
| P value | <0.0001 | <0.0001 | <0.0001 | <0.0001 | <0.0001 | <0.0001 | <0.0001 | <0.0001 | <0.0001 |
| Passed normality test (alpha=0.05)? | No | No | No | No | No | No | No | No | No |
| P value summary | **** | **** | **** | **** | **** | **** | **** | **** | **** |
| | | | | | | | | | |
| **D'Agostino & Pearson test** | | | | | | | | | |
| K2 | 113.1 | 109.1 | 133.2 | 237.9 | 119 | 128.1 | 119 | 113.3 | 119.2 |
| P value | <0.0001 | <0.0001 | <0.0001 | <0.0001 | <0.0001 | <0.0001 | <0.0001 | <0.0001 | <0.0001 |
| Passed normality test (alpha=0.05)? | No | No | No | No | No | No | No | No | No |
| P value summary | **** | **** | **** | **** | **** | **** | **** | **** | **** |
| | | | | | | | | | |
| **Shapiro-Wilk test** | | | | | | | | | |
| W | 0.8136 | 0.8714 | 0.782 | 0.7207 | 0.8648 | 0.8151 | 0.8618 | 0.8604 | 0.8657 |
| P value | <0.0001 | <0.0001 | <0.0001 | <0.0001 | <0.0001 | <0.0001 | <0.0001 | <0.0001 | <0.0001 |
| Passed normality test (alpha=0.05)? | No | No | No | No | No | No | No | No | No |
| P value summary | **** | **** | **** | **** | **** | **** | **** | **** | **** |
| | | | | | | | | | |
| **Kolmogorov-Smirnov test** | | | | | | | | | |
| KS distance | 0.2166 | 0.09765 | 0.1604 | 0.1369 | 0.1144 | 0.1515 | 0.1167 | 0.1227 | 0.1069 |
| P value | <0.0001 | <0.0001 | <0.0001 | <0.0001 | <0.0001 | <0.0001 | <0.0001 | <0.0001 | <0.0001 |
| Passed normality test (alpha=0.05)? | No | No | No | No | No | No | No | No | No |
| P value summary | **** | **** | **** | **** | **** | **** | **** | **** | **** |

**Supplementary figure 3:** Normality of distribution parameters for serum NEFA, and duration of sample storage duration before freezing comparing using 4 different tests. Each test is mentioned in bold in the first column. Each test showed an abnormal distribution

# Supplementary figure -4

## Normality of distribution

| | Adm Lipase | TG 16:0 | TG 16:1 | TG 18:0 | TG 18:1w9 | TG 18:1w7 | TG total C18:1 | TG 18:2 | total TG FA | total TG FA x Adm lipase |
|---|---|---|---|---|---|---|---|---|---|---|
| Compare normal and lognormal | | | | | | | | | | |
| Probability normal (Gaussian) | 0% | 0% | 0% | 0% | 0% | 0% | 0% | 0% | 0% | 0% |
| Probability lognormal | 100% | 100% | 100% | 100% | 100% | 100% | 100% | 100% | 100% | 100% |
| Likelihood ratio (LR) | 2.355E-39 | 1.716E-252 | 2.663E-191 | 0 | 2.266E-245 | 1.745E-194 | 2.55E-243 | 3.662E-244 | 1.662E-249 | 4.104E-244 |
| 1/LR | 4.246E+38 | 5.826E+251 | 3.756E+190 | +infinity | 4.413E+244 | 5.729E+193 | 3.922E+242 | 2.731E+243 | 6.017E+248 | 2.437E+243 |
| Which distribution is more likely? | Lognormal | Lognormal | Lognormal | Lognormal | Lognormal | Lognormal | Lognormal | Lognormal | Lognormal | Lognormal |
| | | | | | | | | | | |
| **Test for normal distribution** | | | | | | | | | | |
| **D'Agostino & Pearson test** | | | | | | | | | | |
| K2 | 69.71 | 561.1 | 413 | 583.2 | 567.9 | 536 | 567.2 | 570.5 | 566.1 | 531.4 |
| P value | <0.0001 | <0.0001 | <0.0001 | <0.0001 | <0.0001 | <0.0001 | <0.0001 | <0.0001 | <0.0001 | <0.0001 |
| Passed normality test (alpha=0.05)? | No | No | No | No | No | No | No | No | No | No |
| P value summary | **** | **** | **** | **** | **** | **** | **** | **** | **** | **** |
| | | | | | | | | | | |
| **Shapiro-Wilk test** | | | | | | | | | | |
| W | 0.8276 | 0.1251 | 0.2843 | 0.07581 | 0.1208 | 0.1889 | 0.1226 | 0.1154 | 0.1203 | 0.1286 |
| P value | <0.0001 | <0.0001 | <0.0001 | <0.0001 | <0.0001 | <0.0001 | <0.0001 | <0.0001 | <0.0001 | <0.0001 |
| Passed normality test (alpha=0.05)? | No | No | No | No | No | No | No | No | No | No |
| P value summary | **** | **** | **** | **** | **** | **** | **** | **** | **** | **** |
| | | | | | | | | | | |
| **Kolmogorov-Smirnov test** | | | | | | | | | | |
| KS distance | 0.1625 | 0.4108 | 0.3573 | 0.4412 | 0.408 | 0.3671 | 0.4066 | 0.4063 | 0.4099 | 0.4146 |
| P value | <0.0001 | <0.0001 | <0.0001 | <0.0001 | <0.0001 | <0.0001 | <0.0001 | <0.0001 | <0.0001 | <0.0001 |
| Passed normality test (alpha=0.05)? | No | No | No | No | No | No | No | No | No | No |
| P value summary | **** | **** | **** | **** | **** | **** | **** | **** | **** | **** |

**Supplementary figure 4:** Normality of distribution for serum lipase, and each fatty acid concentration studied in serum triglycerides (TG), lipase product with TGFAs using 4 different tests. Each test is mentioned in bold in the first column. All tests showed an abnormal distribution.

## Supplementary figure -5

| | TG>500 | TG 301-500 | TG 150-300 | TG< 150 |
|---|---|---|---|---|
| Number of values | 27 | 35 | 100 | 107 |
| | | | | |
| Minimum | 505.3 | 300.8 | 150 | 9.6 |
| 25% Percentile | 561 | 324.6 | 171.7 | 71.6 |
| Median | 730.2 | 369.2 | 199.5 | 104.9 |
| 75% Percentile | 1064 | 425 | 238.8 | 126.9 |
| Maximum | 22234 | 481.4 | 296.9 | 149 |
| Range | 21729 | 180.6 | 146.9 | 139.4 |
| | | | | |
| Mean | 1633 | 373 | 206.7 | 97.65 |
| Std. Deviation | 4133 | 55.62 | 40.99 | 37.68 |
| Std. Error of Mean | 795.4 | 9.402 | 4.099 | 3.643 |

**Supplementary figure 5**: Table describing the characteristics of each AP patient group of serum triglycerides (mentioned at top of each column). The row with the “number of values” depicts the number of patients in each group.

# Supplementary figure -6

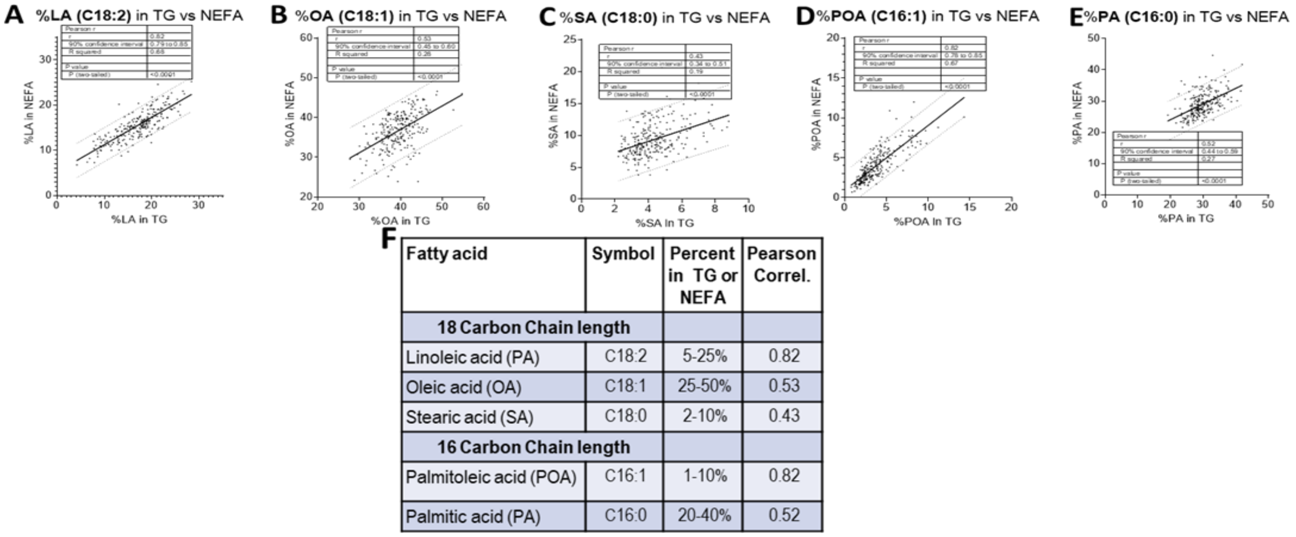


**F**

| Fatty acid | Symbol | Percent in TG or NEFA | Pearson Correl. |
|---|---|---|---|
| **18 Carbon Chain length** | | | |
| Linoleic acid (PA) | C18:2 | 5-25% | 0.82 |
| Oleic acid (OA) | C18:1 | 25-50% | 0.53 |
| Stearic acid (SA) | C18:0 | 2-10% | 0.43 |
| **16 Carbon Chain length** | | | |
| Palmitoleic acid (POA) | C16:1 | 1-10% | 0.82 |
| Palmitic acid (PA) | C16:0 | 20-40% | 0.52 |

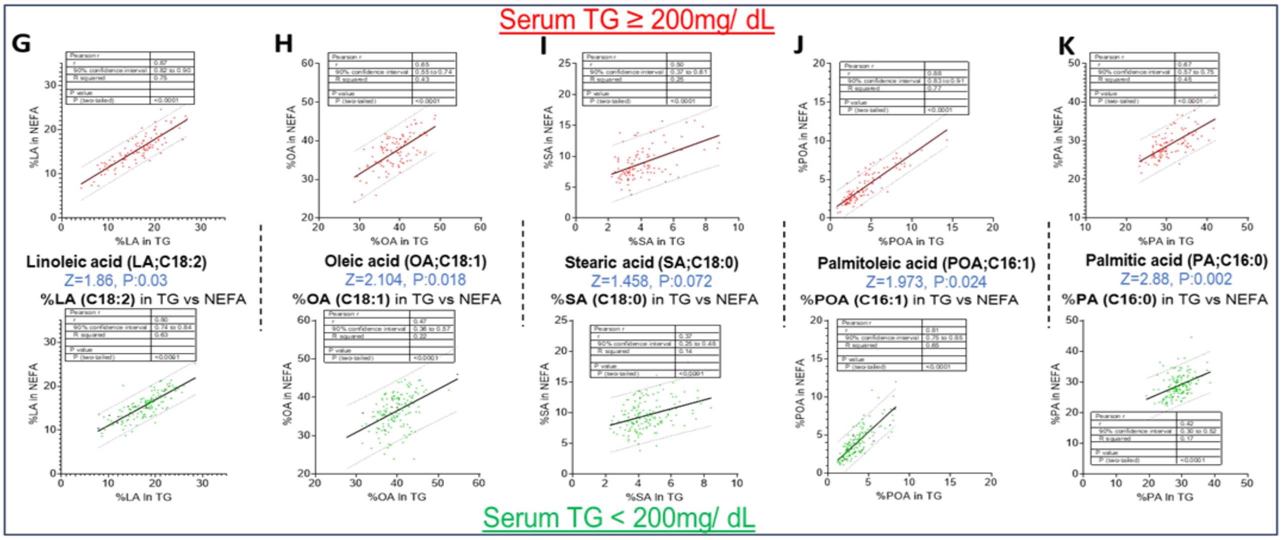


**L**

| Fatty acid | Correl. TG ≥200 | Correl. TG <200 | *P*. value |
|---|---|---|---|
| **18 Carbon Chain length** | | | |
| Linoleic acid (PA) | 0.87 | 0.80 | 0.03 |
| Oleic acid (OA) | 0.65 | 0.47 | 0.018 |
| Stearic acid (SA) | 0.50 | 0.37 | 0.072 |
| **16 Carbon Chain length** | | | |
| Palmitoleic acid (POA) | 0.88 | 0.81 | 0.024 |
| Palmitic acid (PA) | 0.67 | 0.42 | 0.002 |

__Supplementary figure 6:__ **Correlation between % of NEFA in serum and corresponding % TGFAs. A-E:** Graphs showing linear correlations between % of A) Linoleic Acid (LA), B) Oleic Acid (OA), C) Stearic Acid (SA), D) Palmitic Acid (PA) and E) palmitoleic acid (POA) present in serum in the TGFA form (x-axis) and NEFA form ( y-axis). **F:** table showing range of proportions (percentage) for each fatty acid in serum and their Pearson correlations. **G-K** Graphs comparing the above linear correlations for each fatty acid at serum triglycerides ≥ 200mg/dL (upper panel) vs. < 200 mg/dL (lower panel). The text in blue between the panels compares the strength of correlations. **L:** table summarizing the Pearson correlations for each fatty acids at TGs ≥ 200 mg/dL vs. < 200mg/dL, with the last column showing the significance of differences as measured on the Online-Calculator for testing correlations: Psychometrica using the option for comparison of correlations from independent samples.

## Supplementary figure -7

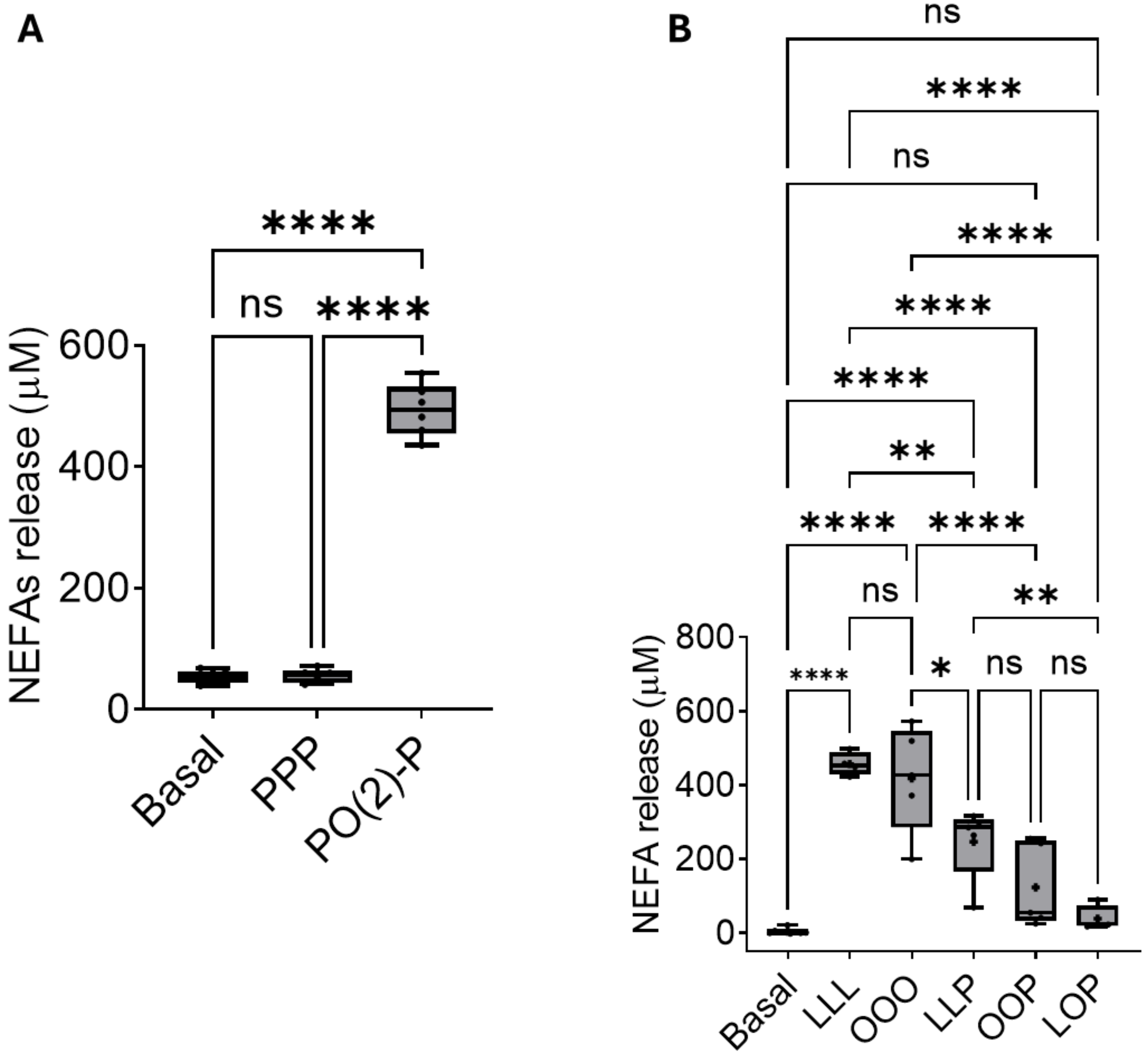


**<u>Supplementary figure 7:</u> A, B:** Box and whisker plots comparing NEFAs released in acinar medium from 300 micromolar triglycerides over 15 minutes. The triglycerides (e.g. LLL) are mentioned below the x-axis. asterisks denoted P values as: “**” for $P<0.01$, “***” for $p< 0.001$, “**** for $P<0.0001$ using an ordinary one-way ANOVA.

# Supplementary figure -8

## Correlation of storage time vs. NEFA concentration

| | Time | Timevs.16:0 | Timevs.16:1 | Timevs.18:0 | Timevs.18:1w9 | Timevs.18:1w7 | Timevs.total C18:1 | Timevs.18:2 | Timevs.total NEFA |
|---|---|---|---|---|---|---|---|---|---|
| Spearman r | | | | | | | | | |
| r | | 0.04931 | 0.05033 | 0.01289 | 0.07435 | 0.05233 | 0.07373 | 0.08866 | 0.07168 |
| 95% confidence interval | | -0.07662 to 0.1737 | -0.07561 to 0.1747 | -0.1127 to 0.1381 | -0.05159 to 0.1980 | -0.07361 to 0.1766 | -0.05221 to 0.1974 | -0.03721 to 0.2118 | -0.05426 to 0.1954 |
| | | | | | | | | | |
| P value | | | | | | | | | |
| P (two-tailed) | | 0.4294 | 0.4199 | 0.8364 | 0.2331 | 0.4017 | 0.237 | 0.1548 | 0.2504 |
| P value summary | | ns | ns | ns | ns | ns | ns | ns | ns |
| **Exact or approximate P value?** | | **Approximate** | **Approximate** | **Approximate** | **Approximate** | **Approximate** | **Approximate** | **Approximate** | **Approximate** |
| **Significant? (alpha = 0.05)** | | **No** | **No** | **No** | **No** | **No** | **No** | **No** | **No** |
| | | | | | | | | | |
| Number of XY Pairs | | 259 | 259 | 259 | 259 | 259 | 259 | 259 | 259 |

**Supplementary figure 8:** Table showing the Spearman correlations (r) and their significance (p) between storage duration and each NEFA.

## Supplementary figure -9

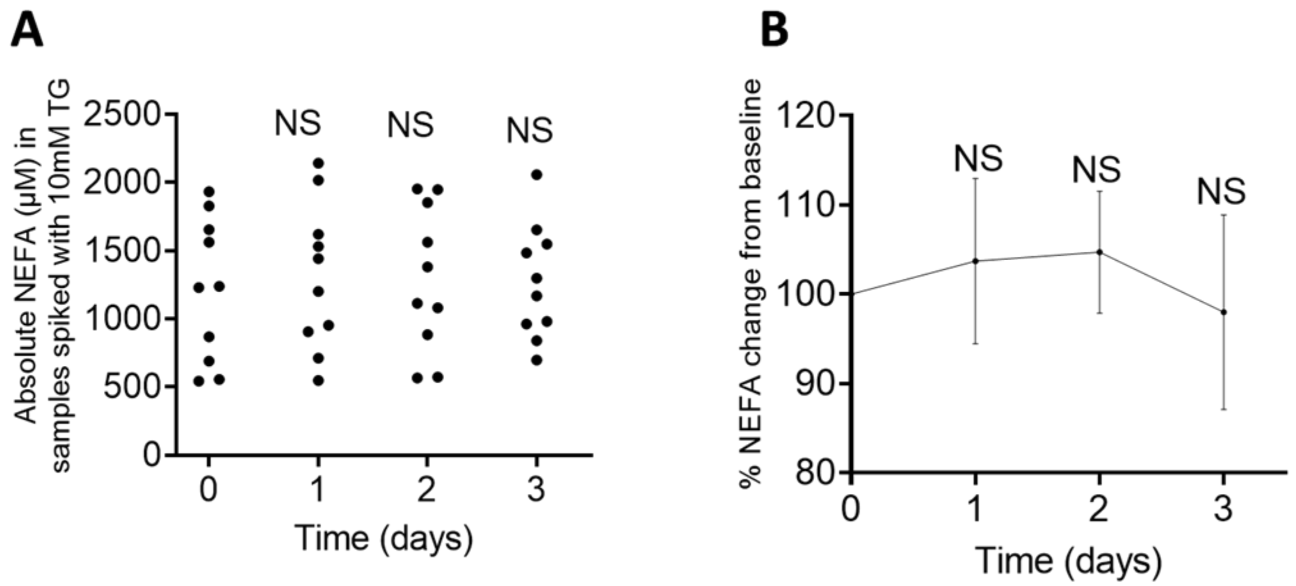


**<u>Supplementary figure 9:</u>** Change in serum NEFA concentrations over 3 days of storage at $4^0$C after adding 10mM triglycerides simulating normal composition as described in methods. **A**: Absolute NEFA concentrations measured immediately after addition of TGs, and daily over the 3 days of storage. **B**: Data shown as % change from baseline. NS: not significant from baseline on an ordinary one-way ANOVA.
